# The Role of Air Pollution and Olfactory Dysfunction in Alzheimer’s Disease Pathogenesis

**DOI:** 10.3390/biomedicines13010246

**Published:** 2025-01-20

**Authors:** Louise Odendaal, Hazel Quek, Carla Cuní-López, Anthony R. White, Romal Stewart

**Affiliations:** 1 Brain and Mental Health, Cellular and Molecular Neurodegeneration, QIMR Berghofer Medical Research Institute, Brisbane, QLD 4006, Australia; louiseodendaal2023@gmail.com (L.O.); hazel.quek@qimrberghofer.edu.au (H.Q.); c.cunilopez@uqconnect.edu.au (C.C.-L.); tony.white@qimrberghofer.edu.au (A.R.W.); 2 UQ Centre for Clinical Research, The University of Queensland, Brisbane, QLD 4006, Australia; 3 School of Biomedical Sciences, Queensland University of Technology, Brisbane, QLD 4059, Australia; 4 School of Biomedical Sciences, The University of Queensland, St Lucia, QLD 4072, Australia; 5 Faculty of Medicine, The University of Queensland, Herston, QLD 4006, Australia

**Keywords:** olfactory system, air pollution, Alzheimer’s disease (AD), neuroinflammation, environmental neurotoxicity, olfactory modelling systems, protein aggregation, public health

## Abstract

The escalating issue of air pollution contributes to an alarming number of premature fatalities each year, thereby posing a significant threat to global health. The focus of recent research has shifted towards understanding its potential association with neurodegenerative diseases, specifically Alzheimer’s disease (AD). AD is recognised for its characteristic deposition of toxic proteins within the brain, leading to a steady deterioration of cognitive capabilities, memory failure, and, ultimately, death. There is burgeoning evidence implying that air pollution may be a contributing factor to this protein build up, thereby intensifying the course of AD. It has been demonstrated that the olfactory system, responsible for smell perception and processing, acts as a potential gateway for airborne pollutants to inflict brain damage. This review aims to elucidate the relationship between air pollution, olfactory deterioration, and AD. Additionally, this review aims to highlight the potential mechanisms through which pollutants might instigate the development of AD and the role of the olfactory system in disease pathogenesis. Moreover, the diverse model systems employed in exploring the correlation, public health policy ramifications, and prospective directions for future research will be discussed.

## 1. Introduction

Air pollution has become a pressing global health issue, contributing to an estimated seven million premature deaths each year [1,2]. Beyond respiratory and cardiovascular impacts, a growing body of research suggests that air pollution may also play a role in the development of neurological diseases, including Alzheimer’s disease (AD) [3]. AD is a progressive neurodegenerative disorder characterised by cognitive decline, memory loss, and, ultimately, mortality [4]. Although the exact aetiology of AD remains elusive, the accumulation of neurotoxic proteins, such as beta-amyloid and tau, is known to be central to its pathogenesis [4]. Emerging evidence suggests that exposure to airborne pollutants may exacerbate the accumulation of these proteins in the brain, potentially accelerating the onset and progression of AD [5].

One potential pathway for pollutant exposure to affect the brain is through the olfactory system, which processes sensory input related to smell. The olfactory system is unique in that it provides a direct link between the external environment and the brain, making it a vulnerable entry point for environmental toxins [6]. Fine particulate matter (PM), which is a major component of air pollution, can be inhaled through the nasal passages and subsequently enter the brain through the olfactory mucosa. This process bypasses the blood–brain barrier, a crucial defence mechanism that typically prevents harmful substances in the bloodstream from reaching brain tissue. Once in the brain, these pollutants can accumulate and induce damage, potentially triggering neuroinflammation and oxidative stress [7]. This exposure pathway underscores the unique susceptibility of the olfactory system to airborne pollutants and highlights a potential route by which air pollution could contribute to AD development.

Research suggests that the olfactory system may be one of the earliest affected regions in AD, with olfactory dysfunction often preceding more recognisable symptoms of cognitive decline [8]. This observation aligns with the hypothesis that pollutants entering the brain through the olfactory pathway could initiate or accelerate AD pathology. For example, airborne PM and associated toxins may trigger inflammation within olfactory structures, which could spread to connected brain areas, leading to the gradual accumulation of beta-amyloid and tau proteins. This mechanism is supported by findings that individuals exposed to high levels of air pollution, particularly in urban environments, exhibit greater deposits of these toxic proteins in the brain compared to those in cleaner environments [3]. Consequently, olfactory dysfunction observed in AD patients may reflect early-stage neural damage initiated by pollutant exposure.

Investigating the link between air pollution and AD requires appropriate model systems to simulate human exposure and disease processes accurately. Animal models, such as rodents exposed to controlled levels of airborne pollutants, have proven useful for studying the progression of neurodegenerative changes related to air pollution. Additionally, in vitro models using human brain organoids allow researchers to explore the molecular and cellular impacts of pollutants on neural tissue. Such models provide insight into how pollutants might directly or indirectly influence the aggregation of beta-amyloid and tau proteins, thereby advancing our understanding of pollution-induced neurotoxicity.

The potential link between air pollution, olfactory dysfunction, and AD emphasises the need for further research to identify the underlying mechanisms and clarify the role of the olfactory pathway in neurodegeneration. Future studies should aim to define how different types of air pollutants affect the brain and explore preventative strategies, particularly in high-risk urban areas. By improving our understanding of the complex relationship between air pollution and AD, we can work toward developing interventions to mitigate the neurotoxic effects of air pollution and potentially reduce the burden of AD and other neurodegenerative diseases.

This review contributes to the field by consolidating research on the links between air pollution, olfactory dysfunction, and AD, a critical yet underexplored area. The field has made substantial progress in identifying air pollution as a significant environmental risk factor for AD, with research highlighting mechanisms such as neuroinflammation, oxidative stress, and BBB disruption. However, these findings remain fragmented, with gaps in understanding the role of specific exposure pathways, such as the olfactory system, and the relative contributions of various pollutants to AD pathogenesis. This review addresses these gaps by integrating evidence from epidemiological studies, animal models, and advanced in vitro systems, providing a comprehensive perspective on how airborne pollutants might accelerate AD pathology. By focusing on the olfactory pathway as a key entry point for neurotoxic pollutants and exploring the interplay between environmental and biological factors, this review offers a unique contribution to the literature. It not only highlights the current state of knowledge but also identifies critical areas for future research, such as the use of cutting-edge experimental models and longitudinal studies, and advocates for public health interventions. Ultimately, this review aims to inform both scientific inquiry and policy initiatives, paving the way for strategies to mitigate the neurotoxic effects of air pollution and reduce the global burden of AD.

## 2. Overview of the Olfactory System

**Figure 1:**
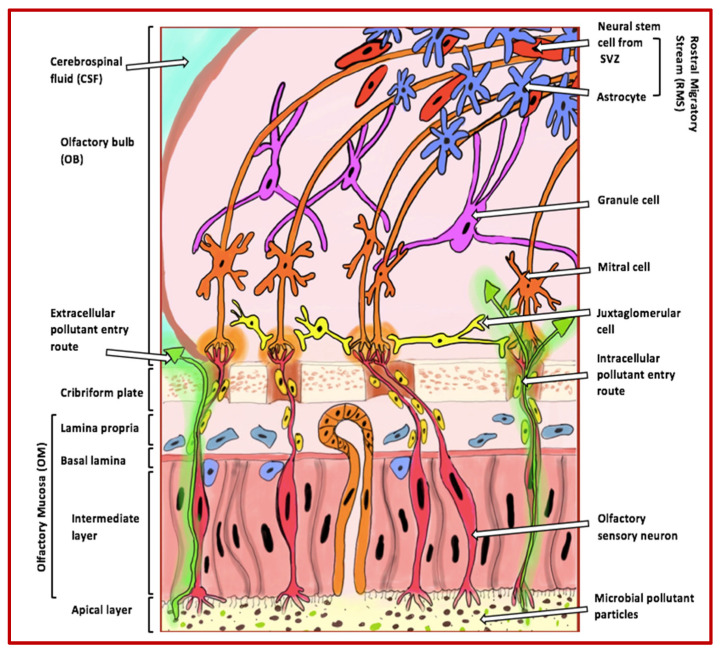
Extracellular and intracellular entry routes via the olfactory mucosa for pollutants into the brain. The figure illustrates how pollutants can enter the brain through extracellular and intracellular routes via the olfactory mucosa. These pollutants, indicated by green arrows, become trapped in the apical layer cilia and can enter the olfactory bulb through the axons of olfactory sensory neurons. Once inside the bulb, the pollutants can move towards and accumulate in higher cognitive areas that are commonly affected in AD. The various cell types of the olfactory bulb that are involved in odour perception include the following: (i) granule cells, which adjust and fine-tune olfactory signals within the olfactory bulb; (ii) mitral cells, the primary projection neurons responsible for conveying processed odour signals to the olfactory cortex; (iii) juxtaglomerular cells, a collection of interneurons that manage activities in glomeruli, assisting in odour differentiation and contrast amplification; (iv) neural stem cells from the rostral migratory stream, which contribute to the continuous replenishment of interneurons; and (v) astrocytes, which provide structural, metabolic, and trophic support to neurons and help maintain the extracellular environment within the olfactory system. Abbreviations: SVZ (subventricular zone).

The olfactory system is responsible for our sense of smell and plays an essential role in our ability to detect and recognise different odours. This system comprises several anatomical structures, including the olfactory mucosa, the olfactory bulbs, and the olfactory cortex [9] (Figure 1).

This highly specialised system allows mammals, including humans, to recognise and process a vast range of odours, with the capability to detect hundreds of thousands of distinct smells being highly conserved across species [10].

At the forefront of the olfactory system is the olfactory mucosa, a specialised tissue located at the top of the nasal cavity. It contains olfactory receptor neurons (ORNs), which detect odorant molecules and transduce them into electrical signals. These neurons are supported by sustentacular cells, which assist in structural support and neuron regeneration [11]. The electrical signals generated by ORNs are transmitted via their axons to the olfactory bulb, a critical structure within the central nervous system (CNS) responsible for initial odour processing. Within the olfactory bulb, sensory signals are processed and systematically organised by glomeruli, which are spatially arranged to facilitate the differentiation and decoding of distinct odours [12]. The glomeruli are populated by various cell types, including mitral cells (primary projection neurons), granule cells (responsible for fine-tuning signals), and juxtaglomerular cells (interneurons involved in odour contrast amplification and differentiation) [11].

Once processed in the olfactory bulb, the information is transmitted to the olfactory cortex and other higher brain regions, including the hippocampus and amygdala, where it is integrated with memory and emotions, allowing for odour recognition and behavioural responses [13]. Recent research has highlighted the advanced capabilities of the olfactory system, such as detecting rapid concentration fluctuations in odour plumes, enabling complex tasks like odour source separation and localisation [14]. Additionally, individual odours can interact in mixtures, either enhancing or suppressing responses to others, demonstrating the system’s sophisticated neural processing capabilities [15].

The olfactory system’s direct exposure to the external environment makes it uniquely vulnerable to pollutants. These pollutants can infiltrate the olfactory mucosa, inducing inflammation and oxidative stress, which damages the mucosa and decreases olfactory function. Studies have shown that exposure to air pollution is linked to a reduced ability to detect odours in older adults [16]. Similarly, animal models demonstrate that diesel exhaust particles cause inflammation and damage to the olfactory epithelium, further emphasising the detrimental impact of pollution on this sensory system [17]. Once pollutants breach the olfactory mucosa, they can reach the olfactory bulb via intracellular and extracellular pathways, bypassing the BBB. This exposure may lead to the accumulation of neurotoxic substances in higher brain regions, such as the hippocampus, that are critical in neurodegenerative diseases such as AD. As such, the olfactory system’s role as both a sensory gateway and a conduit for environmental neurotoxins underscores its importance in studying the progression of diseases like AD [18,19].

## 3. The Composition of Air Pollution

Air pollution is a complex and dynamic mixture of PM, gases, and biological components that significantly affect human health and the environment. It originates from diverse sources, including transportation, industrial activities, agricultural processes, residential heating, and natural phenomena such as wildfires, volcanic eruptions, and dust storms [20,21]. These sources contribute to primary pollutants, directly emitted into the atmosphere, and secondary pollutants, which are formed through atmospheric chemical reactions [22].

**Figure 2:**
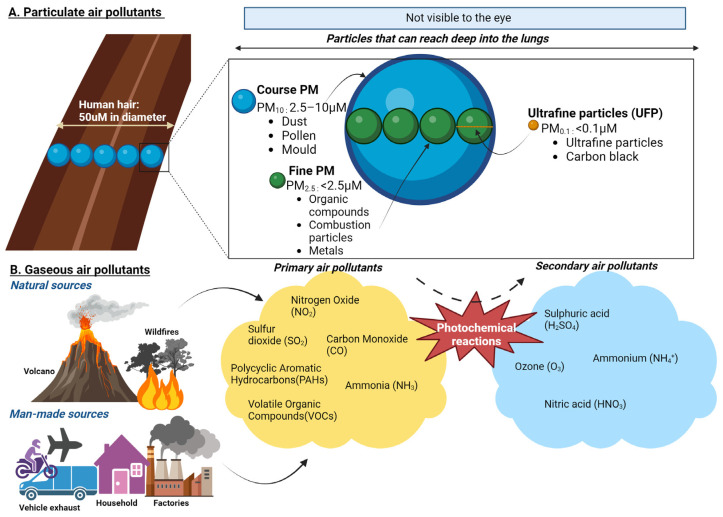
Composition of particulate and gaseous air pollutants. (**A**) PM_10_ (Coarse Particles, 2.5–10 µm): These particles are about 1/5 to 1/20 the diameter of a human hair. Coarse particles are typically inhalable and deposit in the upper airways but are large enough to be visible under a microscope. Common sources include road dust and natural sources like pollen and dust storms. PM_2.5_ (Fine Particles, <2.5 µm): These particles can penetrate deeper into the lungs and enter systemic circulation. They are primarily generated from combustion processes, such as vehicle exhaust, industrial emissions, and wildfires. Ultrafine Particles (UFP, <0.1 µm). These small particles can cross the blood–brain barrier, and enter cells, making them particularly concerning for their health impacts. UFPs are often a by-product of combustion engines and industrial processes. (**B**) Primary air pollutants are directly emitted into the atmosphere from sources such as combustion engines, industrial activities, and natural processes. These include nitrogen dioxide (NO_2_), carbon monoxide (CO), volatile organic compounds (VOCs), and polycyclic aromatic hydrocarbons (PAHs). PAHs are primarily released during the incomplete combustion of organic materials, such as fossil fuels, wood, and biomass. Secondary air pollutants are formed through atmospheric reactions involving primary pollutants and environmental factors such as sunlight and humidity. Key examples include ground-level ozone (O_3_), which forms through photochemical reactions between VOCs and NO_x_ under sunlight, and nitric acid (HNO_3_), produced from the oxidation of nitrogen oxides (NO_x_) in the presence of water vapour. Additional secondary pollutants include particulate matter components such as ammonium nitrates and sulphates, derived from gaseous precursors.

PM, a key component of air pollution, is categorised by size. Coarse particles (PM_10_) deposit primarily in the upper airways, while fine particles (PM_2.5_) and ultrafine particles (<0.1 µm) can penetrate deep into the lungs and enter systemic circulation. PM contains various toxic constituents, including heavy metals such as lead, cadmium, and vanadium; organic pollutants like polycyclic aromatic hydrocarbons; and inorganic substances like nitrates and sulphates [20,23]. Combustion-derived PM, such as diesel exhaust particles, is particularly harmful due to its high oxidative potential, driven by adsorbed metals and organic compounds [20]. Gaseous pollutants, including nitrogen oxides, sulphur dioxide, ozone, and carbon monoxide, are also significant contributors to air pollution and can exacerbate respiratory and cardiovascular diseases [24]. These gases frequently interact with PM to create secondary pollutants such as ammonium nitrates and sulphates [22] (Figure 2). In addition, biological materials, such as pollen, spores, and endotoxins, further aggravate allergic and inflammatory responses in sensitive individuals [20].

PM interacts with biological systems in complex ways. Coarse particles are deposited in the upper airways, while fine and ultrafine particles penetrate deeper into the alveoli and can translocate to organs such as the heart, liver, and brain through the bloodstream or via the olfactory bulb using trans-synaptic mechanisms [20]. The fine and ultrafine particles are particularly concerning due to their high surface-area-to-mass ratio, which facilitates the adsorption of toxic chemicals and enhances their reactivity [25]. Upon inhalation, PM generates reactive oxygen species (ROS), leading to oxidative stress that depletes antioxidants, damages cellular components such as DNA, and activates inflammatory pathways, including MAP kinases and NF-κB [26,27]. These processes are implicated in chronic diseases such as asthma, chronic obstructive pulmonary disease, cardiovascular conditions, and neurodegenerative disorders including AD [23,26].

The composition and health impacts of air pollution vary regionally. Urban areas often experience higher concentrations of combustion-related pollutants like black carbon and nitrogen oxides, primarily from vehicle emissions and industrial activities [22]. Conversely, rural regions may face higher exposure to pollutants from agricultural activities, such as ammonia emissions, and natural sources like dust, wild fires and biomass burning [20]. Vulnerable populations, including children, the elderly, and individuals with pre-existing health conditions, are disproportionately affected by air pollution. Socioeconomic disparities further exacerbate these risks, as low-income communities are often located in highly polluted areas and have limited access to healthcare [28,29].

The systemic effects of PM extend beyond the respiratory system. Once in circulation, PM components can induce cardiovascular dysfunction through endothelial damage, increased coagulation, and plaque formation [30]. Indeed, ultrafine particles can cross the BBB, contributing to neuroinflammation and cognitive decline, particularly in populations exposed to high levels of urban air pollution [23,26]. Efforts to mitigate air pollution exposure must include reducing emissions from transportation, industrial processes, and residential heating. Improved urban planning, adoption of cleaner technologies, and stringent environmental regulations are critical to reducing the health burden of air pollution. Public health initiatives should also prioritise vulnerable populations to address health inequities and ensure equitable health outcomes [20,22,31]. Table 1 summarises the composition of air pollution and its overall impact on health.

## 4. Alzheimer’s Disease and Air Pollution

An increasing body of evidence suggests that air pollution exposure may play a role in the development and progression of neurodegenerative diseases, such as AD, Parkinson’s disease and amyotrophic lateral sclerosis [3,5,34]. Alzheimer’s, in particular, has been extensively studied concerning air pollution. Numerous epidemiological studies have reported a link between air pollution exposure and cognitive decline, dementia, and specifically AD [35]. For example, a large prospective cohort study in the United States found that long-term exposure to PM_2.5_ correlated with accelerated cognitive decline and increased AD risk [36]. Another study in Sweden discovered that traffic-related air pollution exposure was associated with a heightened risk of both AD and vascular dementia [37].

The mechanisms linking air pollution to AD are multifaceted and complex. As mentioned earlier, air pollution is increasingly recognised as a key factor in the development of AD and other neurodegenerative conditions, contributing to these disorders through mechanisms such as neuroinflammation, oxidative stress, and disruption of the BBB [5,38]. Exposure to airborne pollutants, particularly PM_2.5_, initiates the release of pro-inflammatory cytokines and chemokines, which activate microglia and astrocytes within the brain [39]. Chronic activation of these glial cells leads to sustained inflammation and the production of ROS, driving oxidative stress and neuronal damage [40]. This persistent oxidative environment accelerates the aggregation of Aβ plaques and the hyperphosphorylation of tau proteins, which are hallmark features of AD pathology [41]. Below, Table 2 highlights the mechanistic insights into the relationship between air pollution and AD.

**Figure 3:**
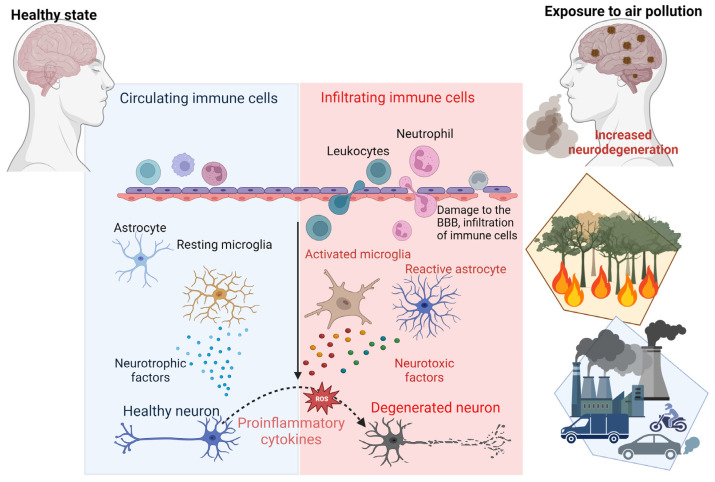
Mechanisms of air-pollution-induced neurodegeneration. This schematic representation highlights the key processes through which air pollution contributes to neurodegenerative diseases such as AD. Exposure to air pollution triggers the production of pro-inflammatory cytokines and chemokines, activating microglia and astrocytes. Chronic activation of microglia and astrocytes generates reactive oxygen species (ROS), leading to neuronal cell damage. Moreover, air pollution can compromise the integrity of the blood–brain barrier (BBB), causing infiltration of immune cells, toxic protein accumulation and inflammation in the brain.

Furthermore, air pollution has been shown to compromise the integrity of the BBB. The weakening of the BBB allows neurotoxic substances and inflammatory mediators to infiltrate the CNS, exacerbating neuronal inflammation and promoting the accumulation of toxic proteins such as Aβ and tau. These processes contribute to the progressive neurodegeneration observed in AD [7]. Importantly, PM_2.5_ can bypass the BBB entirely by entering through the olfactory mucosa, directly exposing the CNS to harmful pollutants [5,43] (Figure 3). Studies have shown that these particles are phagocytosed by mucosal cells in the nasal epithelium and transported to the olfactory bulb and deeper brain regions, creating a direct pathway for pollutants to exacerbate neurodegenerative processes [19].

The role of the olfactory system in facilitating pollutant entry into the brain aligns with the Braak hypothesis, which suggests that certain pathogens or toxic proteins may infiltrate the brain through the nasal cavity and trigger a cascade of neurodegenerative events. In AD, Aβ proteins may act as scaffolding forming aggregates that spread in a prion-like fashion along neural pathways, particularly those linked to the olfactory system. This propagation of pathology to key brain regions is thought to play a central role in disease progression [44]. Experimental models have provided additional evidence supporting this hypothesis [44]. Indeed, transgenic AD mouse models exposed to air pollution demonstrate increased Aβ deposition, tau phosphorylation, and gliosis in regions such as the hippocampus and olfactory bulb [3,34,45].

Epidemiological and experimental data suggest that different components of air pollution may have distinct neurotoxic effects. For instance, black carbon, a marker of traffic-related pollution, is strongly associated with neuroinflammation and oxidative damage, while secondary pollutants such as nitrogen dioxide and ozone contribute to systemic inflammation and cardiovascular risks that indirectly affect the brain [46]. A meta-analysis revealed that regions with high levels of PM_2.5_ exposure exhibited significantly increased rates of AD incidence compared to less polluted areas, with odds ratios as high as 2.20 in heavily polluted regions [46].

Additionally, air pollution has been implicated in epigenetic modifications that may predispose individuals to AD. For example, exposure to airborne pollutants can alter DNA methylation and histone acetylation patterns, promoting transcriptional changes associated with neurodegeneration [34]. These epigenetic alterations may be particularly impactful during critical developmental periods, raising concerns about the lifelong effects of early exposure to air pollution on brain health [42]. Vulnerable populations, including children, the elderly, and individuals with pre-existing health conditions, are disproportionately affected [29]. Limited access to healthcare and environmental mitigation strategies amplifies the burden of pollution-induced neurodegeneration in low-income communities [29,37].

As global urbanisation and industrialisation continue to rise, the public health implications of air pollution’s role in AD are becoming increasingly urgent. Regulatory measures to reduce emissions of PM_2.5_, NO_2_, and other harmful pollutants are critical for mitigating these effects [2,37,47]. Additionally, future research should focus on identifying therapeutic interventions to counteract pollution-induced neurotoxicity, such as antioxidant treatments or anti-inflammatory strategies that target key pathways implicated in AD progression [38,40].

### Olfactory Dysfunction as an Early Marker of Alzheimer’s Disease

Olfactory dysfunction has been suggested as a potential early indicator of AD due to its connection with the regions of the brain affected by the disease, such as the entorhinal cortex and hippocampus, which are crucial for memory and are among the first to deteriorate in AD [48,49,50]. In keeping with this, olfactory dysfunction has also been associated with the progression from mild cognitive impairment to AD [49,51]. Indeed air pollution may contribute to both olfactory dysfunction and AD. Numerous investigations have identified a relationship between air pollution exposure and cognitive decline [52,53]. These studies emphasise the detrimental effects of PM_2.5_ and nitrogen oxide exposure on cognitive functions such as verbal and mathematical abilities. Additional research is required to pinpoint the specific pathways through which air pollution influences cognitive functions and develop targeted interventions to reduce the risk of cognitive decline and neurodegenerative diseases in susceptible populations [31].

Air pollution has also been shown to cause inflammation and oxidative stress, which leads to neuronal damage and cognitive decline [3,5]. Neuroinflammation and oxidative stress are key mechanisms in this process [54,55]. Exposure to air pollutants activates microglia and astrocytes, leading to the release of pro-inflammatory cytokines, such as tumour necrosis factor-alpha (TNF-α), interleukin-1β (IL-1β), and interleukin-6 (IL-6). Chronic activation of these immune cells exacerbates neuronal damage [25,56,57]. Furthermore, pollutants like PM_2.5_ promote the generation of ROS, which overwhelm antioxidant defences, causing lipid peroxidation, DNA damage, and protein misfolding [58,59]. These oxidative modifications impair neuronal function and increase the risk of Aβ and tau protein aggregation, hallmark features of AD [57,60], which have been detected in children with no known risk factors for AD [32]. Additionally, pollutants compromise the BBB, allowing peripheral immune cells and neurotoxic substances to infiltrate the brain, perpetuating inflammation and oxidative stress [19,54,61].

Higher levels of air pollution have also been associated with an increased risk of olfactory dysfunction [37,47,62,63,64]. A study conducted in Mexico City, a location known for high levels of air pollution, found that children exposed to higher pollution levels had lower olfactory function than those in less polluted areas [65]. These results emphasise the need to understand the impact of air pollution on olfactory function and its broader implications for cognitive health and neurodegenerative diseases. Olfactory dysfunction may serve as a valuable marker for early detection of AD, with air pollution exposure potentially contributing to this dysfunction. Future research should investigate the mechanisms connecting air pollution to olfactory dysfunction and explore potential interventions to counteract the effects of air pollution on cognitive function.

## 5. Current Model Systems Used to Elucidate the Links Between Air Pollution, Olfactory Dysfunction, and Alzheimer’s Disease

### 5.1. Animal Models of Air Pollution Exposure and Alzheimer’s Disease

An expanding body of literature has begun to investigate the effects of air pollution on AD pathology by employing animal models. The olfactory system has emerged as a focus for researchers because it may serve as an entry point for air pollution, causing the onset of AD. Experiments on mice exposed to air pollution particles revealed increased beta-amyloid plaque accumulation, neuroinflammation, and oxidative stress in both the olfactory bulb and cortex [3,40]. Moreover, mice exposed to diesel exhaust particles showed increased concentrations of beta-amyloid and tau protein in the hippocampus, a critical region for memory and learning processes, as well as in the olfactory bulb [66]. In line with this, another study showed that mice exposed to concentrated ambient particles had increased beta-amyloid plaque accumulation in both the hippocampus and the olfactory bulb [67]. In addition, a recent report showed that mice exposed to nanoparticle-rich diesel exhaust particles increased oxidative stress and neuroinflammation in the olfactory bulb and increased beta-amyloid plaque accumulation in both the olfactory bulb and the hippocampus [18]. These findings suggest that exposure to air pollution, specifically ultrafine particulate matter and diesel exhaust particles, cause debilitating effects on the olfactory system and hippocampal regions of the brain, which could exacerbate AD pathology. However, while animal models have been highly beneficial in elucidating the effect of air pollution in AD, the findings may not be entirely applicable to humans, as there are significant differences in brain structure and function between species. Hence, future research should incorporate advanced in vitro techniques, such as human brain organoids or induced pluripotent stem cell (iPSC)-derived neurons, along with epidemiological studies and controlled human exposure trials to better understand the direct impact of air pollution on AD pathology and to develop effective strategies for mitigating these effects.

### 5.2. Human Studies of Air Pollution Exposure and Olfactory Function and Alzheimer’s Disease

Researchers have studied the effects of air pollution exposure on olfactory function in individuals from various regions worldwide. The study conducted in the polluted environment of Mexico City showed that children with higher exposure to PM_2.5_ had a significantly worse sense of smell than those with lower exposure levels [65]. A similar study conducted in Taiwan found that exposure to high levels of PM_2.5_ was associated with a decline in olfactory function in elderly individuals [38]. In addition to these observational studies, a few intervention studies have also been conducted to assess the impact of air pollution reduction on olfactory function. For example, a study conducted in Beijing found that after implementing an air pollution control policy, participants’ olfactory function improved significantly [68]. Another study conducted in London found that reduced traffic-related air pollution was associated with improved olfactory function in children [69]. Several mechanisms have been proposed to explain the relationship between air pollution exposure and olfactory dysfunction. One proposed mechanism is that air pollution can cause inflammation and oxidative stress in the olfactory epithelium, damaging olfactory receptor neurons and ultimately impairing olfactory function [7]. Another proposed mechanism is that air pollution can lead to changes in the brain, including neuroinflammation and the accumulation of beta-amyloid plaques, characteristic of AD [5]. These changes in the brain may ultimately lead to olfactory dysfunction and contribute to the development of AD. Indeed, there is growing evidence that olfactory dysfunction may be an early indicator of AD. Studies have found that individuals with AD and those at risk for AD, such as those with mild cognitive impairment, have impaired olfactory function [48,70]. Moreover, studies have suggested that olfactory dysfunction may precede other cognitive symptoms of AD over several years, making it a potential biomarker for early disease detection [48,70].

### 5.3. In Vitro Models of Air Pollution Exposure and Alzheimer’s Disease

**Figure 4:**
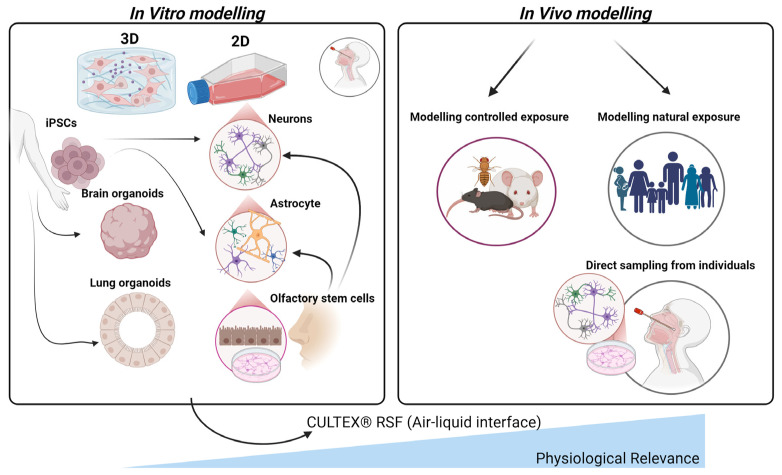
Comparative in vitro and in vivo models for studying air pollution impacts on the olfactory system. This figure illustrates various in vitro (**left**) and in vivo (**right**) approaches to model the effects of air pollution on olfactory tissues. In vitro models, derived from induced pluripotent stem cells (iPSCs), include 2D and 3D cultures such as brain and lung organoids, neurons, astrocytes, and olfactory stem cells, providing controlled environments to study specific cellular responses. As such, lung organoids provide a valuable platform for studying the impact of air-pollution-induced systemic inflammation and oxidative stress, mechanisms that can propagate through the circulatory system and olfactory pathways to the brain, contributing to neuroinflammation and the pathogenesis of AD. The CULTEX^®^ Radial Flow System (RFS) is highlighted for its air–liquid interface, which mimics physiological conditions by allowing direct pollutant exposure to cultured cells. In vivo models comprise controlled exposure studies in animals and natural exposure assessments in human populations, including direct sampling from individuals’ nasal tissues. Together, these models support a comprehensive understanding of air pollution’s effects on the olfactory system and its potential contributions to neurodegenerative disease development.

The importance of in vitro models for studying the effects of air pollution on the olfactory system and AD cannot be understated, particularly in light of ethical concerns and the limitations of extrapolating animal study results to humans (Figure 4). Additionally, the substantial disparities in brain structure and function across species present a challenge in translating biomarkers discovered through animal research to human studies. Various in vitro cell models have been developed for this purpose, which offer a more physiologically relevant environment to study the impact of air pollution on olfactory tissue (Figure 4). Primary cultures derived from human olfactory mucosal cells also provide a relevant model, although challenges such as limited tissue accessibility and donor variability exist [71,72,73,74]. Induced pluripotent stem cells (iPSCs) present a promising alternative for creating olfactory sensory neurons and other olfactory cell types, enabling researchers to study individual-specific responses to air pollution and identify genetic factors affecting susceptibility [75,76]. Additionally, co-culture models with multiple cell types and iPSC-derived brain organoids can help clarify the intricate interactions between various cell types in response to air pollution exposure [77].

A limitation of the previously mentioned model systems is that submerged cultures used for in vitro exposure of cultivated cells to airborne pollutants do not accurately mimic the in vivo environment [78]. This discrepancy is because pollutant deposition in submerged cultures relies on accumulation and Brownian diffusion, which differs from deposition in lung tissues [79]. To overcome this issue, researchers developed the CULTEX^®^ Radial Flow System, which simulates the in vivo environment by cultivating cells at the air–liquid interface and facilitating direct cell–pollutant contact without media interference [80]. This system provides uniform exposure across the entire cell layer and employs suitable cell models for the target tissue type. After cultivation, cells can be exposed to atmospheric pollutants under dynamic or static conditions. Dynamic exposure necessitates a more sophisticated setup, including continuous and homogenous test atmosphere production, reproducible pollutant dispersion via electrostatic precipitation, and constant atmosphere removal [80].

Table 3 provides a summary of the model systems used to investigate the links between air pollution, olfactory dysfunction, and AD.

## 6. Implications for Public Health

Current studies examining the relationship between air pollution and AD have crucial implications for public health policies. Evidence suggests that exposure to air pollution, particularly PM_2.5_, correlates with an elevated risk of AD and other neurodegenerative diseases [36,76]. Epidemiological and animal studies have consistently demonstrated a connection between air pollution and AD risk. For instance, long-term exposure to PM_2.5_ has been shown to raise the likelihood of AD and cognitive decline [36]. Additionally, urban air pollution is particularly linked to a higher risk of AD in genetically predisposed individuals, such as those carrying the APOE-ε4 allele [36]. Given these associations, reducing air pollution could significantly lower the risk of AD. Public health policies should emphasise minimising air pollutant exposure through urban planning measures, such as limiting traffic-related pollution, stricter air quality regulations, and encouraging alternative transportation modes [3]. Furthermore, public health efforts should focus on vulnerable populations, including the elderly and those with genetic predispositions, by offering targeted interventions and communicating risks effectively [30].

Recent research adds to this understanding, indicating that long-term exposure to ambient fine PM_2.5_ and nitrogen dioxide significantly raises dementia and AD risks [34]. Moreover, research has shown how such exposures particularly affect urban areas where pollutant levels exceed recommended thresholds [34]. Additionally, a meta-analysis emphasised this risk further, showing a twofold increase in AD incidence in heavily polluted regions compared to lightly polluted areas [46]. As stated earlier mechanistic studies reveal that PM_2.5_ and ultrafine particles disrupt the BBB, induce neuroinflammation, and promote Aβ aggregation, all of which exacerbate AD pathology [58,60]. Also, animal models, demonstrated that chronic PM_2.5_ exposure increases Aβ deposition and glial activation, highlighting how these processes contribute to disease progression [45].

Public health measures must prioritise reducing air pollutant exposure through stricter emission standards, promotion of green infrastructure, and education on protective measures. Studies have, emphasised the significance of addressing woodsmoke and wildfire exposure, which are major contributors to PM_2.5_ levels in certain regions [83]. Awareness campaigns could inform high-risk populations, such as urban residents and APOE-ε4 carriers, about air pollution risks and protective measures [36]. Future research should explore long-term public health interventions, the interaction between genetic predispositions and environmental exposures, and the effectiveness of policy measures in reducing the burden of air-pollution-induced AD [3,34,36]. Addressing these aspects could lead to significant public health improvements and mitigate the global burden of neurodegenerative diseases such as AD.

## 7. Future Perspectives

Future research should prioritise understanding the intricate connections between air pollution and AD, addressing gaps in mechanistic understanding and public health responses. Longitudinal studies are essential to monitor air pollution exposure and cognitive outcomes over extended periods. These studies should include diverse populations, focusing on urban residents exposed to high levels of PM_2.5_ and individuals with genetic predispositions such as APOE-ε4 carriers. Such cohorts can provide valuable data on how chronic exposure accelerates AD pathogenesis and identify vulnerable populations needing targeted interventions [34,36].

Mechanistic research must delve deeper into how air pollutants interact with the brain’s immune cells, such as microglia and astrocytes, to drive neuroinflammation and oxidative stress [25,40,56]. This process facilitates Aβ aggregation, tau hyperphosphorylation, and neuronal apoptosis, which are hallmarks of AD pathology [5,40].

The development of advanced experimental models will play a pivotal role in elucidating these processes. Current models, while informative, lack the physiological relevance needed to replicate human responses to pollutants. Integrated in vitro systems, such as brain organoids and co-culture models of olfactory epithelial cells and neurons, can provide deeper insights (Table 3). Incorporating technologies like the CULTEX^®^ Radial Flow System allows researchers to simulate air–liquid interface exposure, mimicking real-world conditions more accurately [42,80]. Epidemiological and experimental studies should also explore the role of the olfactory pathway as a primary entry point for airborne pollutants. The olfactory bulb’s unique vulnerability and its connectivity to higher-order brain regions make it a focal point for understanding early neurodegenerative changes. Studies should investigate how pollutants traverse the olfactory mucosa, bypass the BBB, and propagate pathology through prion-like mechanisms involving Aβ and tau proteins [5,34].

Emerging evidence suggests that the nasal microbiome could play a secondary but important role in mediating the effects of air pollution on the brain [84]. Pollutant-induced dysbiosis in the nasal cavity may exacerbate local inflammation and compromise the mucosal barrier, indirectly influencing olfactory dysfunction and neuroinflammation [18,54,84]. Research on the nasal microbiome’s composition and its changes due to chronic pollution exposure could reveal secondary pathways linking environmental factors to AD [19,42,84]. Therapeutic interventions represent a promising area of innovation. Antioxidants, anti-inflammatory agents, and therapies aimed at restoring BBB integrity could mitigate the neurotoxic effects of air pollution. Additionally, microbiome-modulating therapies such as probiotics and prebiotics may help restore microbial balance in the nasal cavity, offering a complementary approach to reducing neuroinflammatory cascades [41,83]. Lastly, public health measures must address the root causes of exposure by implementing stricter air quality regulations and urban planning strategies [29]. Reducing vehicular emissions, increasing green spaces, and promoting cleaner energy sources are critical for lowering exposure to harmful pollutants [37,42,47]. Public awareness campaigns can educate communities about the cognitive risks associated with air pollution and encourage protective measures such as using air purifiers and minimising outdoor activities during high-pollution periods [37,46]. By integrating these research directions, future efforts can deepen our understanding of the links between air pollution and AD, paving the way for effective therapeutic and policy interventions to mitigate the impact of environmental neurotoxins on cognitive health. Below, Table 4 outlines key future directions in air pollution and AD research highlighting critical areas for advancing understanding and intervention strategies.

## 8. Conclusions

This review has provided valuable insights into the complex relationship between air pollution exposure, olfactory impairment, and AD. We have examined the mechanisms through which airborne pollutants may influence the onset and progression of AD, with a particular emphasis on the role of the olfactory system as a critical entry point for neurotoxic particles. The olfactory pathway’s unique vulnerability to environmental toxins highlights its potential role in early neurodegenerative changes that precede symptomatic cognitive decline. Furthermore, we reviewed the use of animal and in vitro models to investigate these associations, which have provided essential data on how pollutants contribute to neuroinflammation, oxidative stress, and the accumulation of toxic proteins such as beta-amyloid and tau in the brain. However, the exact molecular mechanisms by which pollutant particles damage the BBB and induce AD neuropathology remain unclear.

Our exploration underscores that the interplay between environmental and biological factors in AD pathogenesis is a vital area for further study. Understanding how pollutants interact with brain structures could help uncover key mechanisms that initiate or accelerate AD pathology, especially in populations exposed to high levels of air pollution. Moreover, this knowledge has significant implications for public health policies aimed at reducing the prevalence and progression of AD. By highlighting the impact of air quality on brain health, policymakers could advocate for stricter air quality standards and urban planning initiatives that reduce exposure to harmful pollutants. This approach could be particularly beneficial in densely populated urban areas, where pollution levels are highest, and the risks to cognitive health are most pronounced.

The findings in this review also suggest avenues for innovative preventative strategies. For instance, developing therapeutic interventions that protect or repair the olfactory system might mitigate the early neurotoxic effects of pollution. Additionally, as our understanding of the link between pollution and neurodegeneration grows, there is a strong case for public awareness campaigns that educate individuals about the cognitive risks associated with long-term pollution exposure, encouraging personal and community-level actions to minimize exposure where possible.

Future research should prioritise longitudinal studies that track air pollution exposure and cognitive outcomes over time, as well as studies that explore the molecular processes that govern how pollutants cross the olfactory mucosa and accumulate in the brain. Advancing the sophistication of experimental models, such as human brain organoids or more refined animal models, could provide even deeper insights into the pathological progression linked to pollution. Ultimately, by advancing research in this area, we can inform public health interventions that not only address AD prevention but also enhance overall cognitive well-being, aiming toward a future where environmental health is recognised as integral to neuroprotective strategies.

## Figures and Tables

**Table 1:** Summary of air pollution composition and health impacts.

Component	Sources	Key Constituents	Health Impacts	References
**Particulate Matter**	Combustion, industrial processes, natural phenomena	Heavy metals, PAHs, nitrates, sulphates	Respiratory diseases (asthma, COPD), systemic effects (cardiovascular dysfunction). Neurological disorders (neuroinflammation, increase neurodegenerative hallmarks)	[20,23,32,33]
**Gaseous Pollutants**	Vehicles, industry, chemical reactions	NO_2_, SO_2_, O_3_, CO	Respiratory irritation, secondary pollutant formation	[22]
**Biological Materials**	Agriculture, natural sources	Pollens, spores, endotoxins	Exacerbation of allergies, inflammatory responses	[20]
**Fine/Ultrafine Particulate matter**	Combustion, urban pollution	Adsorbed metals and organic compounds	Penetration into bloodstream, systemic inflammation, organ damage	[20,26]
**Regional Variability**	Urban: vehicle emissions, industry; Rural: agriculture, natural sources	Combustion-related pollutants, ammonia	Heightened vulnerability among children, elderly, and low-income communities	[22,28]

**Table 2:** Mechanistic insights into air pollution and Alzheimer’s disease.

Mechanism	Description	References
**Neuroinflammation**	Chronic PM_2.5_ exposure activates microglia and astrocytes, leading to the sustained release of pro-inflammatory cytokines such as IL-1β, TNF-α, and IL-6. This prolonged inflammatory state contributes to neuronal injury and synaptic dysfunction.	[27,39,40]
**Oxidative Stress**	Airborne pollutants generate excessive ROS, depleting antioxidants and causing damage to lipids, proteins, and DNA in the brain. This oxidative stress promotes Aβ aggregation and tau hyperphosphorylation, key pathological features of AD.	[7,40,42]
**Blood–Brain Barrier Disruption**	PM_2.5_ and ultrafine particles weaken BBB integrity, allowing toxic substances and inflammatory mediators to enter the CNS. This breach enhances Aβ plaque accumulation and neuronal apoptosis, worsening AD pathology.	[5,7,42]
**Epigenetic Modifications**	Air pollution may induce DNA methylation changes and histone modifications, leading to transcriptional shifts that heighten susceptibility to AD.	[34,42]

**Table 3:** Summary of model systems for investigating links between air pollution, olfactory dysfunction, and Alzheimer’s disease.

Model System	Details	Advantages	Disadvantages	References
**Animal Models**	- Mice exposed to air pollution showed increased Aβ plaque accumulation, neuroinflammation, and oxidative stress in the olfactory bulb and hippocampus.	- Established link between air pollution and AD-like pathology.	- Limited translatability to humans due to differences in brain structure and function.	[3,40,67]
	- Diesel exhaust particles increased Aβ and tau protein levels in the hippocampus and olfactory bulb.	- Controlled environment allows for systematic study of specific pollutants.	- Ethical concerns related to animal use in research.	[18,66]
	- Ultrafine particulate matter exacerbated AD pathology.			
**Human Studies**	- High PM_2.5_ exposure correlated with reduced olfactory function in children and elderly populations.	- Directly relevant to understanding human health impacts.	- Observational studies prone to confounding factors and variability.	[7,38,65]
	- Air pollution reduction policies improved olfactory function in regions like Beijing and London.	- Provides real-world evidence for the link between air pollution and olfactory dysfunction.	- Longitudinal studies are resource-intensive and may lack precise exposure measurements.	[68,69]
	- Mechanisms include inflammation and oxidative stress in the olfactory epithelium and accumulation of Aβ plaques.			[5]
**In Vitro Models**	- iPSC-derived neurons and brain organoids allow for species-relevant studies of air pollution impacts.	- Highly controllable and physiologically relevant.	- Submerged cultures may not accurately mimic in vivo pollutant exposure.	[75,76,77,81,82]
	- CULTEX^®^ Radial Flow System enables direct pollutant exposure to cells, mimicking in vivo conditions.	- Reduces reliance on animal models, addressing ethical concerns.	- Requires specialised equipment and expertise.	[80]
	- Primary human olfactory mucosal cell cultures offer physiological relevance despite challenges like tissue accessibility.	- Provides insights into individual susceptibility to pollutants.	- Donor variability and limited tissue availability.	[71,72,73,74]

**Table 4:** Key future directions in air pollution and Alzheimer’s disease research.

Focus Area	Details	References
**Longitudinal Studies**	Track air pollution exposure and cognitive decline over time, focusing on urban and genetically at-risk populations.	[34,36]
**Mechanistic Insights**	Explore pollutant interactions with microglia, astrocytes, and the BBB to identify molecular targets for mitigating neuroinflammation and oxidative stress.	[5,40,42]
**Experimental Models**	Use advanced in vitro systems like brain organoids and the CULTEX^®^ Radial Flow System to replicate real-world exposure and cellular responses.	[42,80]
**Olfactory Pathway Research**	Investigate how pollutants traverse the olfactory mucosa, bypass the BBB, and propagate pathology via prion-like mechanisms.	[5,19,34]
**Nasal Microbiome**	Examine how dysbiosis influences olfactory dysfunction and neuroinflammation and evaluate microbiome-based therapeutic strategies.	[19,42,83]
**Therapeutic Development**	Develop interventions such as antioxidants, anti-inflammatory agents, and BBB-strengthening treatments to counteract pollution-induced neurotoxicity.	[40,41,83]
**Public Health Policies**	Implement stringent air quality regulations, urban planning to reduce emissions, and public awareness campaigns highlighting the cognitive risks of air pollution.	[5,37,46]

## Data Availability

Not applicable.

## References

[1] Lelieveld J., Klingmüller K., Pozzer A., Pöschl U., Fnais M., Daiber A., Münzel T. (2019). Cardiovascular disease burden from ambient air pollution in Europe reassessed using novel hazard ratio functions. Eur. Heart J..

[2] Owusu P.A., Sarkodie S.A. (2020). Global estimation of mortality, disability-adjusted life years and welfare cost from exposure to ambient air pollution. Sci. Total Environ..

[3] Block M.L., Elder A., Auten R.L., Bilbo S.D., Chen H., Chen J.-C., Cory-Slechta D.A., Costa D., Diaz-Sanchez D., Dorman D.C. (2012). The outdoor air pollution and brain health workshop. Neurotoxicology.

[4] Selkoe D.J., Hardy J. (2016). The amyloid hypothesis of Alzheimer’s disease at 25 years. EMBO Mol. Med..

[5] Block M.L., Calderón-Garcidueñas L. (2009). Air pollution: Mechanisms of neuroinflammation and CNS disease. Trends Neurosci..

[6] Chin-Chan M., Navarro-Yepes J., Quintanilla-Vega B. (2015). Environmental pollutants as risk factors for neurodegenerative disorders: Alzheimer and Parkinson diseases. Front. Cell. Neurosci..

[7] Calderón-Garcidueñas L., Engle R., Mora-Tiscareño A., Styner M., Gómez-Garza G., Zhu H., Jewells V., Torres-Jardón R., Romero L., Monroy-Acosta M.E. (2011). Exposure to severe urban air pollution influences cognitive outcomes, brain volume and systemic inflammation in clinically healthy children. Brain Cogn..

[8] Attems J., Walker L., Jellinger K.A. (2015). Olfaction and aging: A mini-review. Gerontology.

[9] Sharma A., Kumar R., Aier I., Semwal R., Tyagi P., Varadwaj P. (2019). Sense of Smell: Structural, Functional, Mechanistic Advancements and Challenges in Human Olfactory Research. Curr. Neuropharmacol..

[10] Lane G., Zhou G., Noto T., Zelano C. (2020). Assessment of direct knowledge of the human olfactory system. Exp. Neurol..

[11] Witt M. (2020). Anatomy and Development of the Human Gustatory and Olfactory Systems.

[12] Wang I.-H., Murray E., Andrews G., Jiang H.-C., Park S.J., Donnard E., Durán-Laforet V., Bear D.M., Faust T.E., Garber M. (2022). Spatial transcriptomic reconstruction of the mouse olfactory glomerular map suggests principles of odor processing. Nat. Neurosci..

[13] Mackay-Sim A. (2012). Olfactory mucosa: Neural stem and progenitor cells for nervous system repair and cell models of brain disease. Progenitor and Stem Cell Technologies and Therapies.

[14] Ackels T., Erskine A., Dasgupta D., Marin A.C., Warner T.P., Tootoonian S., Fukunaga I., Harris J.J., Schaefer A.T. (2021). Fast odour dynamics are encoded in the olfactory system and guide behaviour. Nature.

[15] Xu L., Li W., Voleti V., Zou D.-J., Hillman E.M., Firestein S. (2020). Widespread receptor-driven modulation in peripheral olfactory coding. Science.

[16] Scussiatto H.O., Da Silva J.L.B., Figueiredo A.F., Ramos R.A.M.R., de Rezende Pinna F., Voegels R.L., Pinto J.M., Fornazieri M.A. (2023). Association of air pollution with olfactory identification performance of São Paulo residents: A cross-sectional study. Int. Arch. Occup. Environ. Health.

[17] Yokota S., Hori H., Umezawa M., Kubota N., Niki R., Yanagita S., Takeda K. (2013). Gene expression changes in the olfactory bulb of mice induced by exposure to diesel exhaust are dependent on animal rearing environment. PLoS ONE.

[18] Ehsanifar M., Montazeri Z., Taheri M.A., Rafati M., Behjati M., Karimian M. (2021). Hippocampal inflammation and oxidative stress following exposure to diesel exhaust nanoparticles in male and female mice. Neurochem. Int..

[19] Lucchini R., Dorman D., Elder A., Veronesi B. (2012). Neurological impacts from inhalation of pollutants and the nose–brain connection. Neurotoxicology.

[20] Falcon-Rodriguez C.I., Osornio-Vargas A.R., Sada-Ovalle I., Segura-Medina P. (2016). Aeroparticles, composition, and lung diseases. Front. Immunol..

[21] Laumbach R.J., Kipen H.M. (2012). Respiratory health effects of air pollution: Update on biomass smoke and traffic pollution. J. Allergy Clin. Immunol..

[22] Bourdrel T., Bind M.-A., Béjot Y., Morel O., Argacha J.-F. (2017). Cardiovascular effects of air pollution. Arch. Cardiovasc. Dis..

[23] Ghio A.J., Carraway M.S., Madden M.C. (2012). Composition of air pollution particles and oxidative stress in cells, tissues, and living systems. J. Toxicol. Environ. Health Part B.

[24] Sagheer U., Al-Kindi S., Abohashem S., Phillips C.T., Rana J.S., Bhatnagar A., Gulati M., Rajagopalan S., Kalra D.K. (2024). Environmental Pollution and Cardiovascular Disease: Part 1 of 2: Air Pollution. JACC Adv..

[25] Song J., Han K., Wang Y., Qu R., Liu Y., Wang S., Wang Y., An Z., Li J., Wu H. (2022). Microglial activation and oxidative stress in PM_2.5_-induced neurodegenerative disorders. Antioxidants.

[26] Schraufnagel D.E. (2020). The health effects of ultrafine particles. Exp. Mol. Med..

[27] Cuní-López C., Ng M.F., Stewart R., Milton L.A., Etebar F., Sun Y., Vivian E., Nguyen T.H., Asare P.F., Lupton M.K. (2024). Impact of wildfire smoke and diesel exhaust on inflammatory response in aging human microglia. bioRxiv.

[28] Pope III C.A., Dockery D.W. (2006). Health effects of fine particulate air pollution: Lines that connect. J. Air Waste Manag. Assoc..

[29] Tshehla C., Wright C.Y. (2019). 15 Years after the national environmental management air quality Act: Is legislation failing to reduce air pollution in South Africa?. S. Afr. J. Sci..

[30] Kioumourtzoglou M.-A., Schwartz J.D., Weisskopf M.G., Melly S.J., Wang Y., Dominici F., Zanobetti A. (2016). Long-term PM_2.5_ exposure and neurological hospital admissions in the northeastern United States. Environ. Health Perspect..

[31] Mohammadzadeh M., Khoshakhlagh A.H., Grafman J. (2024). Air pollution: A latent key driving force of dementia. BMC Public Health.

[32] Calderón-Garcidueñas L., Solt A.C., Henríquez-Roldán C., Torres-Jardón R., Nuse B., Herritt L., Villarreal-Calderón R., Osnaya N., Stone I., García R. (2008). Long-term air pollution exposure is associated with neuroinflammation, an altered innate immune response, disruption of the blood-brain barrier, ultrafine particulate deposition, and accumulation of amyloid beta-42 and alpha-synuclein in children and young adults. Toxicol. Pathol..

[33] Shi L., Wu X., Danesh Yazdi M., Braun D., Abu Awad Y., Wei Y., Liu P., Di Q., Wang Y., Schwartz J. (2020). Long-term effects of PM(2·5) on neurological disorders in the American Medicare population: A longitudinal cohort study. Lancet Planet. Health.

[34] Shi L., Steenland K., Li H., Liu P., Zhang Y., Lyles R.H., Requia W.J., Ilango S.D., Chang H.H., Wingo T. (2021). A national cohort study (2000–2018) of long-term air pollution exposure and incident dementia in older adults in the United States. Nat. Commun..

[35] Genc S., Zadeoglulari Z., Fuss S.H., Genc K. (2012). The adverse effects of air pollution on the nervous system. J. Toxicol..

[36] Cacciottolo M., Wang X., Driscoll I., Woodward N., Saffari A., Reyes J., Serre M.L., Vizuete W., Sioutas C., Morgan T.E. (2017). Particulate air pollutants, APOE alleles and their contributions to cognitive impairment in older women and to amyloidogenesis in experimental models. Transl. Psychiatry.

[37] Oudin A., Forsberg B., Adolfsson A.N., Lind N., Modig L., Nordin M., Nordin S., Adolfsson R., Nilsson L.-G. (2016). Traffic-related air pollution and dementia incidence in northern Sweden: A longitudinal study. Environ. Health Perspect..

[38] Ajmani G.S., Suh H.H., Pinto J.M. (2016). Effects of ambient air pollution exposure on olfaction: A review. Environ. Health Perspect..

[39] Li N., Sioutas C., Cho A., Schmitz D., Misra C., Sempf J., Wang M., Oberley T., Froines J., Nel A. (2003). Ultrafine particulate pollutants induce oxidative stress and mitochondrial damage. Environ. Health Perspect..

[40] Levesque S., Taetzsch T., Lull M.E., Kodavanti U., Stadler K., Wagner A., Johnson J.A., Duke L., Kodavanti P., Surace M.J. (2011). Diesel exhaust activates and primes microglia: Air pollution, neuroinflammation, and regulation of dopaminergic neurotoxicity. Environ. Health Perspect..

[41] Peters A. (2023). Ambient air pollution and Alzheimer’s disease: The role of the composition of fine particles. Proc. Natl. Acad. Sci. USA.

[42] Calderón-Garcidueñas L., Stommel E.W., Rajkumar R.P., Mukherjee P.S., Ayala A. (2021). Particulate air pollution and risk of neuropsychiatric outcomes. What we breathe, swallow, and put on our skin matters. Int. J. Environ. Res. Public Health.

[43] Oberdörster G., Elder A., Rinderknecht A. (2009). Nanoparticles and the brain: Cause for concern?. J. Nanosci. Nanotechnol..

[44] Walker L.C. (2018). Prion-like mechanisms in Alzheimer disease. Handb. Clin. Neurol..

[45] Sahu B., Mackos A.R., Floden A.M., Wold L.E., Combs C.K. (2021). Particulate matter exposure exacerbates amyloid-β plaque deposition and gliosis in APP/PS1 mice. J. Alzheimer’s Dis..

[46] Fu P., Yung K.K.L. (2020). Air pollution and Alzheimer’s disease: A systematic review and meta-analysis. J. Alzheimer’s Dis..

[47] Oudin A., Segersson D., Adolfsson R., Forsberg B. (2018). Association between air pollution from residential wood burning and dementia incidence in a longitudinal study in Northern Sweden.(Research Article)(Clinical report). PLoS ONE.

[48] Devanand D.P., Lee S., Manly J., Andrews H., Schupf N., Masurkar A., Stern Y., Mayeux R., Doty R.L. (2015). Olfactory identification deficits and increased mortality in the community. Ann. Neurol..

[49] Devanand D.P., Liu X., Tabert M.H., Pradhaban G., Cuasay K., Bell K., de Leon M.J., Doty R.L., Stern Y., Pelton G.H. (2008). Combining Early Markers Strongly Predicts Conversion from Mild Cognitive Impairment to Alzheimer’s Disease. Biol. Psychiatry.

[50] Mesholam R.I., Moberg P.J., Mahr R.N., Doty R.L. (1998). Olfaction in neurodegenerative disease: A meta-analysis of olfactory functioning in Alzheimer’s and Parkinson’s diseases. Arch. Neurol..

[51] Park J.-W., Kwon D.-Y., Choi J.H., Park M.-H., Yoon H.-K. (2018). Olfactory dysfunctions in drug-naive Parkinson’s disease with mild cognitive impairment. Park. Relat. Disord..

[52] Power M.C., Kioumourtzoglou M.-A., Hart J.E., Okereke O.I., Laden F., Weisskopf M.G. (2015). The relation between past exposure to fine particulate air pollution and prevalent anxiety: Observational cohort study. BMJ.

[53] Weuve J., Puett R.C., Schwartz J., Yanosky J.D., Laden F., Grodstein F. (2012). Exposure to particulate air pollution and cognitive decline in older women. Arch. Intern. Med..

[54] Ehsanifar M., Banihashemian S., Ehsanifar M. (2021). Exposure to air pollution nanoparticles: Oxidative stress and neuroinfl ammation. J. ISSN.

[55] Kuntić M., Hahad O., Münzel T., Daiber A. (2024). Crosstalk between Oxidative Stress and Inflammation Caused by Noise and Air Pollution—Implications for Neurodegenerative Diseases. Antioxidants.

[56] Gómez-Budia M., Konttinen H., Saveleva L., Korhonen P., Jalava P.I., Kanninen K.M., Malm T. (2020). Glial smog: Interplay between air pollution and astrocyte-microglia interactions. Neurochem. Int..

[57] Kang Y.J., Tan H.Y., Lee C.Y., Cho H. (2021). An air particulate pollutant induces neuroinflammation and neurodegeneration in human brain models. Adv. Sci..

[58] Calderón-Garcidueñas L., Herrera-Soto A., Jury N., Maher B.A., González-Maciel A., Reynoso-Robles R., Ruiz-Rudolph P., van Zundert B., Varela-Nallar L. (2020). Reduced repressive epigenetic marks, increased DNA damage and Alzheimer’s disease hallmarks in the brain of humans and mice exposed to particulate urban air pollution. Environ. Res..

[59] Wang L., Wei L.Y., Ding R., Feng Y., Li D., Li C., Malko P., Syed Mortadza S.A., Wu W., Yin Y. (2020). Predisposition to Alzheimer’s and age-related brain pathologies by PM_2.5_ exposure: Perspective on the roles of oxidative stress and TRPM2 channel. Front. Physiol..

[60] Thiankhaw K., Chattipakorn N., Chattipakorn S.C. (2022). PM_2.5_ exposure in association with AD-related neuropathology and cognitive outcomes. Environ. Pollut..

[61] Huang X., Hussain B., Chang J. (2021). Peripheral inflammation and blood–brain barrier disruption: Effects and mechanisms. CNS Neurosci. Ther..

[62] Andersson J., Oudin A., Nordin S., Forsberg B., Nordin M. (2022). PM_2.5_ exposure and olfactory functions. Int. J. Environ. Health Res..

[63] Cao Z., Yang A., White A.J., Purdy F., Li C., Luo Z., D’Aloisio A.A., Suarez L., Deming-Halverson S., Pinto J.M. (2023). Ambient air pollutants and olfaction among women 50–79 years of age from the sister study. Environ. Health Perspect..

[64] Ekström I.A., Rizzuto D., Grande G., Bellander T., Laukka E.J. (2022). Environmental air pollution and olfactory decline in aging. Environ. Health Perspect..

[65] Calderón-Garcidueñas L., Mora-Tiscareño A., Ontiveros E., Gómez-Garza G., Barragán-Mejía G., Broadway J., Chapman S., Valencia-Salazar G., Jewells V., Maronpot R.R. (2008). Air pollution, cognitive deficits and brain abnormalities: A pilot study with children and dogs. Brain Cogn..

[66] Ehsanifar M., Tameh A.A., Farzadkia M., Kalantari R.R., Zavareh M.S., Nikzaad H., Jafari A.J. (2019). Exposure to nanoscale diesel exhaust particles: Oxidative stress, neuroinflammation, anxiety and depression on adult male mice. Ecotoxicol. Environ. Saf..

[67] Fonken L.K., Xu X., Weil Z.M., Chen G., Sun Q., Rajagopalan S., Nelson R.J. (2011). Air pollution impairs cognition, provokes depressive-like behaviors and alters hippocampal cytokine expression and morphology. Mol. Psychiatry.

[68] Guan W.-J., Zheng X.-Y., Chung K.F., Zhong N.-S. (2016). Impact of air pollution on the burden of chronic respiratory diseases in China: Time for urgent action. Lancet.

[69] Kelly F.J., Fussell J.C. (2015). Air pollution and public health: Emerging hazards and improved understanding of risk. Environ. Geochem. Health.

[70] Murphy C. (2019). Olfactory and other sensory impairments in Alzheimer disease. Nat. Rev. Neurol..

[71] Féron F., Perry C., McGrath J.J., Mackay-Sim A. (1998). New techniques for biopsy and culture of human olfactory epithelial neurons. Arch. Otolaryngol. Head Neck Surg..

[72] Rantanen L.M., Bitar M., Lampinen R., Stewart R., Quek H., Oikari L.E., Cunί-Lόpez C., Sutharsan R., Thillaiyampalam G., Iqbal J. (2022). An Alzheimer’s Disease Patient-Derived Olfactory Stem Cell Model Identifies Gene Expression Changes Associated with Cognition. Cells.

[73] Stewart R., Kozlov S., Matigian N., Wali G., Gatei M., Sutharsan R., Bellette B., Kijas A.W., Cochrane J., Coulthard M. (2013). A patient-derived olfactory stem cell disease model for ataxia-telangiectasia. Hum. Mol. Genet..

[74] Stewart R., Wali G., Perry C., Lavin M.F., Féron F., Mackay-Sim A., Sutharsan R. (2017). A patient-specific stem cell model to investigate the neurological phenotype observed in ataxia-telangiectasia. ATM Kinase: Methods and Protocols.

[75] Leeson H.C., Hunter Z., Chaggar H.K., Mackay-Sim A., Wolvetang E.J. (2021). Reprogramming of human olfactory neurosphere-derived cells from olfactory mucosal biopsies of a control cohort. Stem Cell Res..

[76] Xiong S., Zhang S., Guan L., Chen J., Tu X., Li Q., Jiang H. (2017). Differentiation of induced pluripotent stem cells for future olfactory repair using an indirect co-culture technique. Int. J. Clin. Exp. Pathol..

[77] Alfaro-Moreno E., Nawrot T.S., Vanaudenaerde B.M., Hoylaerts M.F., Vanoirbeek J.A., Nemery B., Hoet P.H. (2008). Co-cultures of multiple cell types mimic pulmonary cell communication in response to urban PM_10_. Eur. Respir. J..

[78] Rach J., Budde J., Möhle N., Aufderheide M. (2014). Direct exposure at the air–liquid interface: Evaluation of an in vitro approach for simulating inhalation of airborne substances. J. Appl. Toxicol..

[79] Fröhlich E., Salar-Behzadi S. (2014). Toxicological Assessment of Inhaled Nanoparticles: Role of in Vivo, ex Vivo, in Vitro, and in Silico Studies. Int. J. Mol. Sci..

[80] Aufderheide M., Halter B., Möhle N., Hochrainer D. (2013). The CULTEX RFS: A Comprehensive Technical Approach for the In Vitro Exposure of Airway Epithelial Cells to the Particulate Matter at the Air-Liquid Interface. BioMed Res. Int..

[81] Stewart R., Yan K., Ellis S.A., Bishop C.R., Dumenil T., Tang B., Nguyen W., Larcher T., Parry R., Sng J.D.J. (2023). SARS-CoV-2 omicron BA. 5 and XBB variants have increased neurotropic potential over BA. 1 in K18-hACE2 mice and human brain organoids. Front. Microbiol..

[82] Yan K., Dumenil T., Stewart R., Bishop C.R., Tang B., Nguyen W., Suhrbier A., Rawle D.J. (2024). TMEM106B-mediated SARS-CoV-2 infection allows for robust ACE2-independent infection in vitro but not in vivo. Cell Rep..

[83] Schuller A., Montrose L. (2020). Influence of woodsmoke exposure on molecular mechanisms underlying Alzheimer’s disease: Existing literature and gaps in our understanding. Epigenetics Insights.

[84] Mazumder M.H.H., Hussain S. (2024). Air-Pollution-Mediated Microbial Dysbiosis in Health and Disease: Lung–Gut Axis and Beyond. J. Xenobiotics.

